# Multiple gamma rhythms carry distinct spatial frequency information in primary visual cortex

**DOI:** 10.1371/journal.pbio.3001466

**Published:** 2021-12-21

**Authors:** Chuanliang Han, Tian Wang, Yi Yang, Yujie Wu, Yang Li, Weifeng Dai, Yange Zhang, Bin Wang, Guanzhong Yang, Ziqi Cao, Jian Kang, Gang Wang, Liang Li, Hongbo Yu, Chun-I Yeh, Dajun Xing

**Affiliations:** 1 State Key Laboratory of Cognitive Neuroscience and Learning & IDG/McGovern Institute for Brain Research, Beijing Normal University, Beijing, China; 2 Beijing Institute of Basic Medical Sciences, Beijing, China; 3 Vision Research Laboratory, Center for Brain Science Research and School of Life Sciences, Fudan University, Shanghai, China; 4 Department of Psychology, National Taiwan University, Taipei, Taiwan, China; McGill University, CANADA

## Abstract

Gamma rhythms in many brain regions, including the primary visual cortex (V1), are thought to play a role in information processing. Here, we report a surprising finding of 3 narrowband gamma rhythms in V1 that processed distinct spatial frequency (SF) signals and had different neural origins. The low gamma (LG; 25 to 40 Hz) rhythm was generated at the V1 superficial layer and preferred a higher SF compared with spike activity, whereas both the medium gamma (MG; 40 to 65 Hz), generated at the cortical level, and the high gamma HG; (65 to 85 Hz), originated precortically, preferred lower SF information. Furthermore, compared with the rates of spike activity, the powers of the 3 gammas had better performance in discriminating the edge and surface of simple objects. These findings suggest that gamma rhythms reflect the neural dynamics of neural circuitries that process different SF information in the visual system, which may be crucial for multiplexing SF information and synchronizing different features of an object.

## Abstract 2

Gamma rhythms in many brain regions are thought to play a role in information processing. This study reports the surprising coexistence of three narrow-band gamma rhythms in visual cortex with distinct coding properties for visual features and distinct neural origins.

## Introduction

The gamma rhythm (30 to 100 Hz) is an oscillatory pattern of neural activity. It is commonly found in many brain regions [1–6] and is thought to play an important role in cognitive functions such as learning [7], memory [8,9], and attention [10–13]. Abnormal gamma band activities have been related to mental illness [14–17].

In the visual cortex, visual stimuli with a high degree of spatial frequency (SF) information such as bars [18–21] or contours [14–16] have been found to induce synchronization in the gamma band. However, more recent studies have also demonstrated that stimuli with low SF information, such as large uniform surfaces, also induce strong gamma synchronous responses [22–24]. The finding that both high and low SFs can generate a gamma rhythm is puzzling because high SF information, such as edges [25,26], is thought to be processed by different neural mechanisms than low SF components, such as surfaces [27–31]. There are 2 possible explanations for this contradiction. The first is that a single gamma rhythm can integrate high and low SF information. The second is that distinct gamma rhythms carry different aspects of SF information. The second hypothesis is plausible because distinct narrowband gamma oscillations have been found in cats, primates, and humans [9,20,32–35], among other species. However, the relationship between SF selectivity and specific gamma frequency bands remains poorly understood [36,37].

In this study, we examined how SF information is encoded through gamma band neural activity. Surprisingly, we found 3 distinct narrowband gamma rhythms in the visual cortex with different SF preferences and selectivity. We investigated the response properties and neural origins of these gamma rhythms.

## Materials and methods

### Preparation of anesthetized cats

All procedures were in accordance with the National Institutes of Health Guidelines, and the research protocol was approved by the Biological Research Ethics Committee of Beijing Normal University (ID: IACUC(BNU)-NKLCNL 2017–06) and Fudan University (ID: 2021JS0087). Twelve hours before the experiment, cats were injected with dexamethasone (0.4 mg/kg, subcutaneously). The cats (weighing 2 to 4.5 kg each) were initially anesthetized with isoflurane (5% concentration) and injected with atropine sulfate (0.05 mg/kg, subcutaneously). After we conducted a tracheotomy, the animals were artificially ventilated. During recordings, anesthesia and paralysis were maintained with propofol (2 to 6 mg/kg/h) and vecuronium bromide (0.1 mg/kg/h), respectively. The end tidal CO2 was maintained at 3.5% to 4%, and the body temperature was set at 36.5°C to 37.5°C. The eyes of the cats were treated with 1% atropine sulfate solution to dilate the pupils, and they were fitted with appropriate contact lenses with artificial pupils (1-mm diameter) to restrict the amount of luminance and ensure focus on a tangent screen. Ventilation pressure, heart rate, electrocardiogram, and blood oxygen were monitored continuously throughout the experiment. To maintain the health of the cats prior to the experiment, they were injected with dexamethasone antibiotic every day (0.4 mg/kg) and with atropine sulfate (0.05 mg/kg) every other day. One craniotomy (10 to 15 mm in diameter) was performed over the primary visual cortex (VI; centered at P0–2 and L0, mainly in Brodmann’s area 18 (A18)), and another (approximately 6 mm in diameter) was performed over the lateral geniculate nucleus (LGN) (centered at L8, A9).

### Electrophysiological recordings

Multielectrode arrays (Utah Array, Blackrock Microsystems, USA) were used to record signals in A18 from the horizontal direction (parallel to the cortical surface) in 12 cats. The arrays consisted of 6*8 or 10*10 grids of microelectrodes (1-mm electrode length and 400-micron electrode separation). They were inserted 0.5 to 0.6 mm into the cortex using a pneumatic insertion device. Multielectrode linear arrays (Blackrock Microsystems) were used to record signals in area 18 from the vertical direction (perpendicular to the cortical surface) in another 3 cats. The linear arrays consisted of 24 microelectrodes (spaced 0.1 mm apart, each 0.015 mm in diameter, Plexon, USA). They were inserted 1 to 1.2 mm into the cortex using an automatic controlled insertion device. The multielectrode linear arrays (Plexon) were also used to record signals in the LGN of 6 cats (in 4 of these, recordings were simultaneously conducted in the V1 using Utah arrays). They were inserted 13 to 14 mm into the cortex using an automatic controlled insertion device. To prevent pulsation, the arrays and the exposed cortex were covered with 1.5% to 2% agar. Signals were amplified using a Cerebus 128-channel system (Blackrock Microsystems). All local field potential (LFP) signals were sampled at 500 Hz per channel with a wide-band front-end filter (0.3 to 500 Hz). LFP data were postprocessed by removing channels that were deemed nonfunctional because of broken electrodes or noise.

### Visual stimulation

Visual stimuli were generated using a computer with a Leadtek GeForce 6800 video card and displayed on a cathode-ray tube (CRT) monitor (Sony CPD-G520, mean luminance of 32 cd/m^2^, 1024*768 resolution, refresh rate 100 Hz) placed 40 cm away from the animal’s eyes. Luminance nonlinearities were corrected using the luminance meter (photoresearch PR670). Stimuli were presented only to the eye contralateral to the craniotomy. For each experiment, we first mapped the receptive fields (RFs) of the multiunit activity (MUA) responses using short instances of sparse visual noise and then conducted an experiment with drifting gratings and large squares with uniform black/white surface luminance.

### RF mapping

After manually mapping the RFs of the recorded channels, we used sparse visual noise to precisely locate the RF center. Specifically, a white or black square (0.5 to 2 degrees of visual angle) was flashed on a gray background at different positions in a pseudorandom sequence (usually on a 13 × 13 or 11 × 11 sample grid). The stimuli were presented with an effective frame rate of 100 Hz so that each sparse noise image appeared for 40 ms. Each square was presented 30 to 60 times. The sequence was cut into small segments based on the trial length. This process enabled us to generate a two-dimensional map for each channel. The averaged responses from the x-axis and y-axis of each map were fitted with a one-dimensional Gaussian function to estimate the center position and radius of the RF (σ of the Gaussian function).

### Gratings and uniform stimuli

We first presented drifting gratings with different SFs (approximately 0.01 to 2.5 cycles per degree, 10 conditions) and directions (−18° to 180°, 18 conditions). The stimulus contrast was 90%, and the temporal frequency (TF) was 4 cycles per second. Afterward, all drifting gratings were enlarged (approximately 38°) to cover the RFs of all recording sites, and the stimuli were shown only to the contralateral eye. The temporal sequence of the visual experiment was prestimulus (0.4 seconds), on stimulus (2 seconds), and off stimulus (0.4 seconds). In 2 cats, a large uniform square stimulus (20° for one cat and 25° for the other) (black or white against a gray background) was then presented at different positions, so that the center of the RF for that site fell into the border (edge) or the center (surface) of the stimulus.

### Laminar alignment

To align the different probe placements in terms of depth, we used the laminar pattern of the MUA responses to the visual stimuli with different SFs. Because the thickness of the cortex and verticality of the probe differed between the probe placements, we used the relative depth (ReD) [38,39] to represent the cortical position of each recording site. The ReD is the normalized cortical depth, ranging from 0 to 1. Three variables were used to calculate the ReD. First, the recording site with the lowest SF selectivity was considered to be in the middle of the input layer (Cha1) and was assigned a ReD value of 0.5. Second, the cortical surface was considered to be located 0.050 mm above the uppermost channel (Cha2) with significant visually driven spiking responses (signal-to-noise ratio [SNR] > 3 and the 3 consecutive channels below Cha2 were also expected to meet the criterion of SNR > 3) and was assigned a ReD value of 0. Third, the boundary separating the gray and white matter was considered to be located 0.050 mm below the deepest channel (Cha3) with significant visually driven spiking responses (SNR > 3 and the 3 consecutive channels above Cha3 were also expected to meet the criterion of SNR > 3) and was assigned a ReD value of 1. After the 3 signatures (Cha1, Cha2, and Cha3) had been determined, they were used as references when calculating the ReD for each channel. ReD values of 0.375 to 0.625 were assigned as the input layer of A18 in the cat V1 according to the procedure used in previous studies [40,41].

### Power spectrum analysis

For each trial, the power spectrum of the LFP response over the period 300 to 2,000 ms after stimulus onset (yielding the stimulus power spectrum) was estimated using the multitaper method [42,43] (time-bandwidth product, 3; tapers, 5; Chronux toolbox (http://chronux.org/)), which was implemented using custom software written in MATLAB. Essentially, the multitaper method attempts to reduce the variance of spectral estimates by premultiplying the data with several orthogonal tapers known as Slepian functions. The frequency decomposition of multitapered data segments therefore provides a set of independent spectral estimates that, once averaged, provides an ensemble estimate that is more reliable for noisy data.

### Coherence analysis

We evaluated spike-field coherence (SFC) by calculating the coherency Cxy between different sites (x and y) as the cross-spectra between signals in x and y (Sxy), normalized by the geometric mean of their autospectra (Sxx and Syy) [8]. This was estimated using the multitaper method [44] (time-bandwidth product, 3; tapers, 5; Chronux toolbox (http://chronux.org/)) and implemented using custom software written in MATLAB. To rule out the possibility that the effect of the SFC was solely the effect of the gamma power, we conducted a control analysis in which we calculated the SFC between spike trains, shuffled relative to the LFP signals for each trial within each stimulus condition.

### Model fitting and evaluation

To quantify the responses of the different gamma components (GCs) in terms of different SFs, we used a two-dimensional descriptive model to fit the power spectrum. One dimension was the oscillatory frequency, and the other dimension was the SF.


$$Power(SF,f)=Baseline(SF,f)+\sum_{i=1}^{3}{Comp}_{i}(SF,f)$$ (1)

$$Baseline(SF,f)=\left[\frac{k(SF)}{f^{a(SF)}+b(SF)}\right]+c(SF)$$ (2)

$${Comp}_{i}(SF,f)=W_{i}(SF)∙\exp\left(-\frac{{\left(f-\mu_{i}(SF)\right)}^{2}}{2\sigma_{i}^{2}(SF)}\right)$$ (3)

$$\mu_{i}(SF)=K_{0,i}+K_{i}∙\frac{{SF}^{n_{i}}}{{SF}^{n_{i}}+{SF}_{0}^{n_{i}}}$$ (4)

$$W_{i}(SF)=A_{1,i}∙\exp\left(-\frac{{SF}^{2}}{2\sigma_{1,i}^{2}}\right)-A_{2,i}∙\exp\left(-\frac{{SF}^{2}}{2\sigma_{2,i}^{2}}\right)(A_{1,i}\geq 0;A_{2,i}\geq 0)$$ (5)

We used a two-dimensional descriptive model (1 to 5) to fit the power spectrums of the LFP and MUA for different SFs. The LFP power in the gamma range could be modeled by a baseline and the weighted sum of the 3 frequency components (1). The frequency profile of the baseline was modeled as a 1/f function that decreased monotonically according to frequency (2), and the TF profiles of the 3 components were all modeled as Gaussian functions (3). The peak frequency *μ* of each GC was modeled as sigmoid function (4). The SF profile for the power *W* of the GC was modeled as the difference between the Gaussian function and the sigmoid function for the peak frequency (5). The power spectrums in the edge or surface condition were fitted using formula (1), and the SF tuning curve for the MUA and baseline power in the 3 gamma frequency bands were fitted by formula (5). The goodness of fit was defined as formula (6). Only the sites with a high goodness of fit (larger than 0.8) were analyzed:

$$Goodness\;of\;fit=1-\frac{\sum_{1}^{n}{{(R}_{data}(i)-R_{model}(i))}^{2}}{\sum_{1}^{n}{\left(R_{data}(i)-\frac{\sum_{1}^{n}R_{data}(i)}{n}\right)}^{2}}$$ (6)

We fitted power spectrums with the descriptive model (described by formula 1 with details in formulas 2 to 5) by minimizing the mean square error (MSE) between the model prediction and the data (power spectrum) using the fmincon function in MATLAB. The parameters that depend on SF include *μ_i_* and *W_i_*, and the parameters independent of SF include a, b, c, k, *σ_i_*, SF_0_, n_i_, K_0,i_, K_i_, A_1,i_, A_2,i_, σ_1,i_, and σ_2,i_ (formulas 4 to 5). Instead of determining the number of GCs before model fitting, we fixed the number of GCs at 3 for model fitting to spectrums from all recorded sites. After model fitting, we counted the number of GCs required to explain the power spectrums from each site based on the SNR of each fitted GC. The SNR of each GC was defined as the max weight of the GC (formula 5) divided by the mean power in the corresponding gamma band under the blank condition. If the SNR of a GC was larger than 3, we counted this as a required GC, and otherwise it was considered a negligible GC. In this way, we were able to determine whether a power spectrum had 0, 1, 2, or 3 GCs.

### Decoding the edge and surface of a square

We built a logistic regression model to decode the location within a stimulus (edge or surface) that drove a single site (or group of sites) in a given trial, either by gamma power or mean firing rate. The population response matrix was defined as X, X = (X_1_, …, X_3N_)^T^, where X_i_ was a vector (m by 1, m was the total number of trials) of the neural data (either gamma power or MUA response), and the gamma power was the power of the j^th^ recording site in the low gamma (LG) (i = 3j), medium gamma (MG) (i = 3j + 1), and high gamma (HG) (i = 3j + 2) rhythms, respectively. *N* was the total number of sites included for decoding. As a control, we built a logistic decoder based on the MUA responses using the same procedure. To keep the size of the response matrix (X) constant, we divided the MUA responses from the j^th^ recording site into 3 time bins (if i = 3j, mean rate 300 to 900 ms after the stimulus onset; if i = 3j + 1, mean rate 900 to 1,500 ms after the stimulus onset; and if i = 3j + 2, mean rate 1,500 to 2,000 ms after the stimulus onset). We aggregated all data together for the decoding operation. The units in the X were either all edge response or all surface response. The logistic regression model used to fit the population response to the square location was as follows:

$$P(location\;is\;edge|X)=\frac{1}{1+e^{XW+w0}}$$ (7)

Here, W is a vector (3N by 1) of weights. When *p* ≥ 0.5, the population is predicted to be driven by the stimulus edge; when *p* < 0.5, the population is predicted to be driven by the stimulus surface. We trained the decoder by optimizing the weight matrix (W) to minimize the loss function (formula 8).


$$J=-\frac{1}{m}(\sum_{i=1}^{m}\left(Y_{i}ln(P)+(1-Y_{i})ln(1-P)\right))+\frac{\lambda }{2m}|W|$$ (8)

Here, Y is an M by 1 vector representing the location of the stimulus, where 1 is the edge and 0 is the surface. We used an L1 regularization term to minimize the obtained weights and prevent overfitting. We random separate the dataset into a training set (60%), validation set (15%), and test set (25%). For a regularization parameter (λ), we chose the option that gave the lowest MSE in the cross-validation test among 20 lambda values that were equally spaced (logarithmically) between 0.001 and 40. We used the fminunc function in MATLAB to train the decoder. The results shown here (Fig 3C, S7 Fig) were obtained with the regularization parameter set at 0.1 for all sites.

### Site selection

The inclusion criterion for a site was as follows. The firing rate or gamma power of the site at its preferred SF was significantly higher than that of the baseline (i.e., SNR ratio larger than 3). For the MUA responses, the SNR was defined as the standard deviation of the firing rate for visual stimuli divided by that in the blank condition. For LFPs, the SNR was defined as the standard deviation of the gamma power for visual stimuli divided by the standard deviation of the power in the corresponding gamma frequency band in the blank condition.

### Statistical analysis

We used an independent sample *t* test with the Bonferroni correction to compare the SF selectivity across the 3 GCs, gamma power in the edge and surface conditions, the MUA responses in the edge and surface conditions, and the SNRs of the 3 GCs.

We used the bootstrapping procedure to test the influence of location (in the vertical direction in V1) on the peak power of the 3 gamma rhythms. For the i^th^ bootstrap out of 10,000 iterations (bootstraps, i = 1^…^10,000), we used the following procedure. We randomly selected 12 recording sessions in the original dataset (recording sessions *N* = 12) with the same sessions selected more than once allowed. We then computed the mean locations of the peak power for the 3 gamma rhythms (M_L_ for LG, M_M_ for MG, and M_H_ for HG). After 10,000 repeats, we retrieved 10,000 mean values for the locations with peak power. We tested the differences between M_L_, M_M_, and M_H_ in a pair-wise manner according to the null hypothesis that X would be no larger than Y. The *p*-value was calculated as the proportion of the 10,000 repeats in which X was larger than Y.

## Results

**Fig 1:**
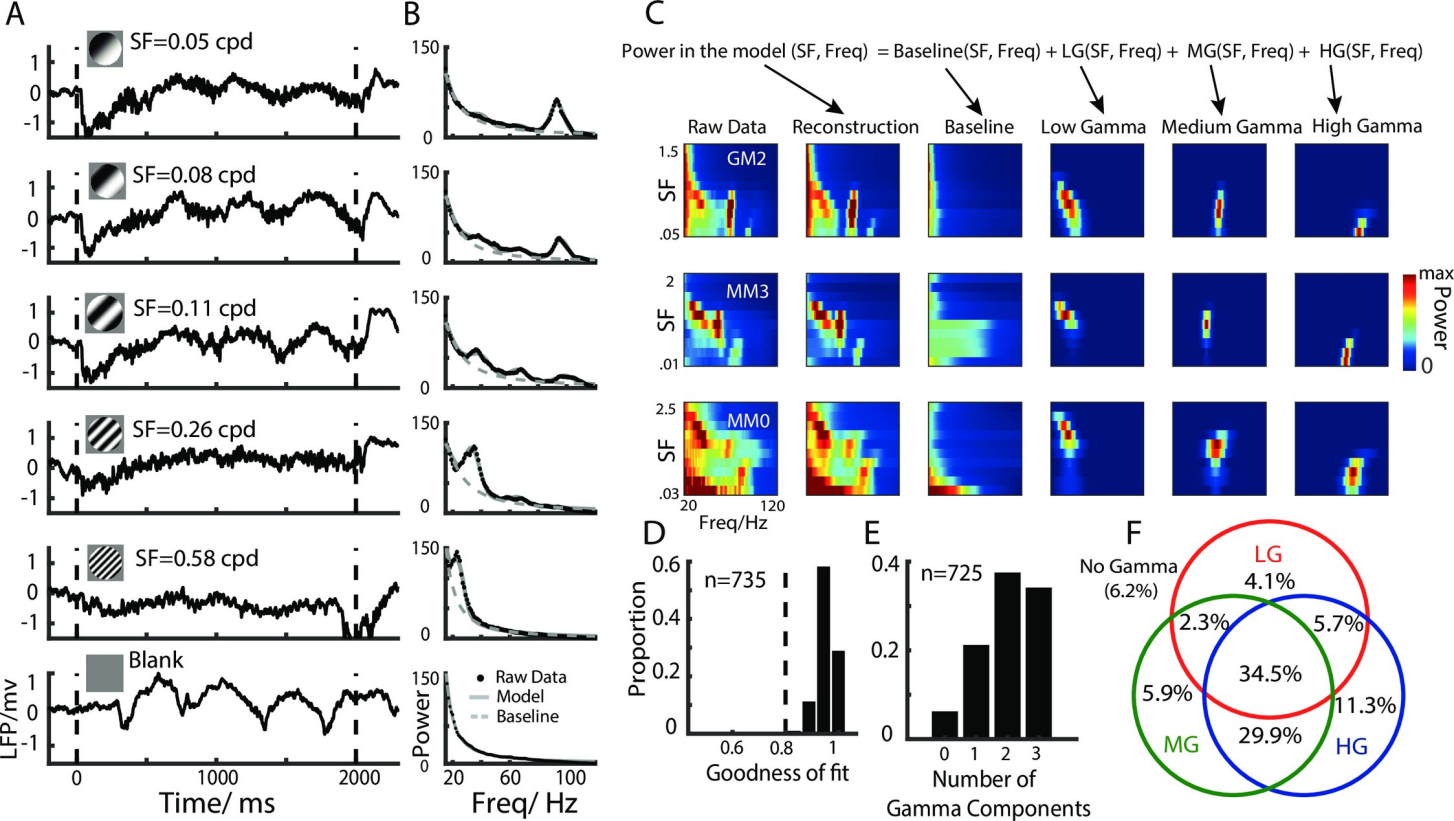
Emergence of multiple gamma rhythms induced at different SFs, explained by the two-dimensional descriptive model. A shows the LFPs elicited by drifting grating patches with different SFs. The dashed black lines indicate the onset and offset of the visual stimulus. The black dots in B show the raw power spectrums of the LFPs for different SFs, and the fitted curves are plotted as grey lines. The dashed grey line shows the fitted baseline generated using the descriptive model. C shows 3 examples of data fitting using the two-dimensional descriptive model. These 3 example sites are from 3 different cats. The left column shows the two-dimensional raw power spectrum data. The horizontal label gives the oscillatory frequency ranging from 20 to 120 Hz, the vertical label gives the SF ranging from 0.03 to 2.5 cpd, and the color denotes the power. The second column on the left shows the reconstructed data analyzed using the descriptive model, which consists of a baseline (the third column on the left) and 3 gamma components (3 columns on the right). D shows the distribution of goodness of fit for this model for all recording sites. E and F show the proportion and number of different gamma types for all recording sites with good fit. The dataset underlying this figure can be found in https://github.com/CleveHan/Gamma-Oscillation. HG, high gamma; LFP, local field potential; LG, low gamma; MG, medium gamma; SF, spatial frequency.

We used multichannel arrays (96 or 48 channels, spaced 400 um apart, Utah array, Blackrock Microsystems) to record MUA and LFP signals in V1 while presenting large sinusoidal drifting gratings (>38° of visual angle, see Materials and methods for more details). Fig 1A shows typical LFP waveforms recorded in response to drifting gratings with different SFs. For most recording sites, drifting gratings were able to induce strong gamma band activities under a range of SFs (Fig 1B).

### Three distinct gamma rhythms in the visual cortex

Surprisingly, we found that visual stimuli at certain SFs could simultaneously induce 3 narrowband gamma rhythms in V1 (Fig 1B). This was consistent in different cats (Fig 1C). To dissect and quantify the distinct narrowband gamma rhythms, we built a descriptive model to explain the power spectrum at different TFs induced by different SFs (Fig 1C). We assumed that the LFP power spectrum could be modeled by the summation of a baseline and 3 bell-shaped components peaking at different TF ranges: LG (25 to 45 Hz), MG (45 to 65 Hz), and HG (65 to 100 Hz). The frequency profiles of the 3 oscillatory components (GCs) were all modeled as Gaussian functions, and the frequency profile of the baseline was modeled as a 1/f function that decreased monotonically in frequency. We further assumed that the GCs would change with the SF of the visual stimulus. Accordingly, the amplitudes of the 3 oscillatory components were modeled as the difference of Gaussian functions [45–48], and their peak frequencies were modeled using the Naka–Rushton function [49–52] (see Materials and methods for the descriptive model and model fitting).

The power spectrums of the LFPs for individual sites could be well reconstructed by this model (see examples of model fit in Fig 1B and 1C). We found that the LFP could be well explained (goodness of fit > 0.8) by the model for 98.6% of the sites (*n* = 725, Fig 1D), and among these, 34.5% of the sites (*n* = 250) had 3 strong (SNR > 3) GCs, 37.9% (*n* = 275) had 2, 21.4% (*n* = 155) had 1, and 6.2% (*n* = 45) had no significant gamma rhythms (Fig 1F, S1 Fig). The 3 gamma rhythms could also be observed in the MUA power spectrum (S2 Fig) and were well fitted by this descriptive model (S3 Fig). After successfully dissecting the 3 GCs, we next demonstrated that the 3 gamma rhythms had distinct response properties in V1.

### The 3 gamma rhythms had distinct SF selectivity

**Fig 2:**
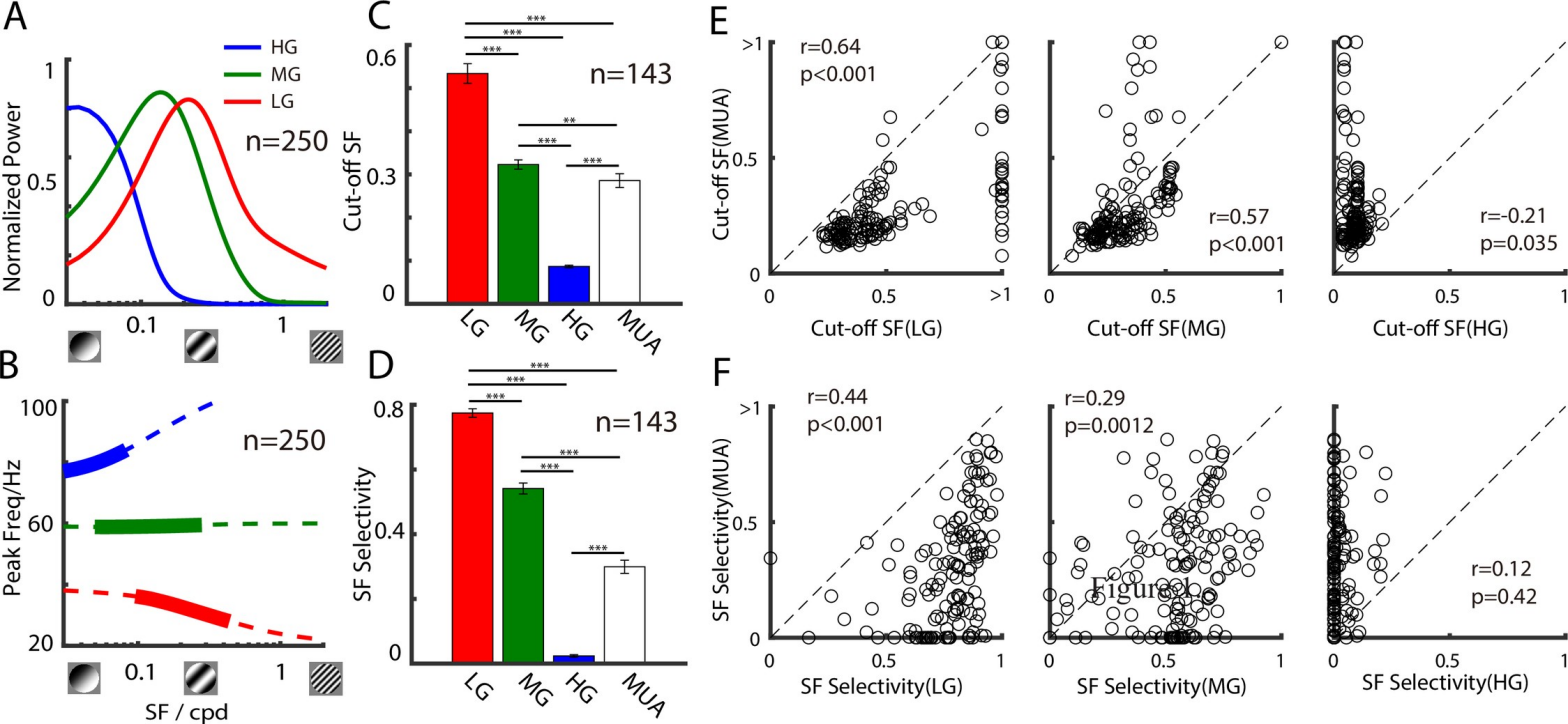
Distinct SF selectivity of 3 gamma rhythms in V1. A shows the SF tuning curves for the power of 3 gamma components (red curve for LG, green curve for MG, and blue curve for HG, 250 sites in 12 animals). The dashed lines in B show the SF tuning curves for the peak frequency of the 3 gamma components estimated by the model. The thick lines denote frequency range estimated by our data (red curve for LG, green curve for MG, and blue curve for HG). C and D show bar graphs of SF selectivity and the cutoff SFs of the 3 gamma components and MUA (143 sites). Scatter plots in E show the relationship between the cutoff SF of the gamma components and MUA (for LG, MG, and HG, respectively). Scatter plots in F show the relationship between the SF selectivity of the gamma components and MUA (for LG, MG, and HG, respectively). The dataset underlying this figure can be found in https://github.com/CleveHan/Gamma-Oscillation. HG, high gamma; LG, low gamma; MG, medium gamma; MUA, multiunit activity; SF, spatial frequency; V1, primary visual cortex.

We found that the power and peak frequency of the 3 gamma rhythms were tuned to different ranges of SF (see Fig 2A and 2B for sites with all 3 GCs, *n* = 250). This could be quantified according to the significant differences in the cutoff SF (quantified as the largest SF corresponding to 50% of the peak value) and SF selectivity (quantified as 1 minus the ratio between the response at a low SF and the response at an optimal SF) among the 3 GCs. Specifically, the HG was low pass (cutoff SF: 0.09 ± 0.003; SF selectivity: 0.02 ± 0.004; mean ± standard error), whereas the MG and LG preferred relatively higher SFs (cutoff SF: 0.33 ± 0.02 for MG and 0.68 ± 0.05 for LG; SF selectivity = 0.54 ± 0.01 for MG and 0.77 ± 0.02 for LG) (see Fig 2C and 2D for sites with all 3 GCs and a good MUA response, *n* = 143). Similar results were found when sites with 2 and 1 gamma peaks were included (S4 Fig).

We next examined how the SF tuning properties of the 3 GCs differed from those of spike activity. We found that both the SF selectivity and cutoff SF for spike activity (MUA) were significantly higher than those of HG (*p* < 0.001, paired *t* test) and lower than those of LG (*p* < 0.001, paired *t* test) or MG (*p* < 0.01, paired *t* test) activity (Fig 2C–2F). This suggests that SF information carried by the mean firing rate at a given recording site has less diversity and a lower spatial resolution than SF information carried by all 3 GCs together. To test whether the diverse SF information we observed in the 3 GCs was also carried by dynamic spike activity [53], we calculated the phase locking strength between spikes and LFPs driven by different stimulus SFs (S5 Fig). We found that the MUA was phase locked to the LFP in the LG range when driven by a stimulus with high SF and phase locked in the HG range when driven by a stimulus with a low SF. In summary, diverse SF information was not only multiplexed by the 3 gamma bands, but also carried by neural dynamic responses that locked to the phases of the 3 GCs.

We also observed different correlations between spike activity (MUA) and the 3 narrowband GCs in terms of their SF tunings: The cutoff SF of MUA was positively correlated to that of LG (r = 0.64, *p* < 0.001) and MG (r = 0.57, *p* < 0.001), but the correlation between the cutoff SF of HG and that of MUA was weak (r = −0.21, r = 0.035) (Fig 2E). A similar result was obtained for the relationship between the SF selectivity of gamma activation and MUA (LG, r = 0.44, *p* < 0.001; MG, r = 0.29, p = 0.0012; HG, r = 12, p = 0.42) (Fig 2F). These results indicate that the 3 GCs may have different neural originations, which we address in a later section.

### Synchronization of the gamma rhythms differentiated SF-related features of a simple object

**Fig 3:**
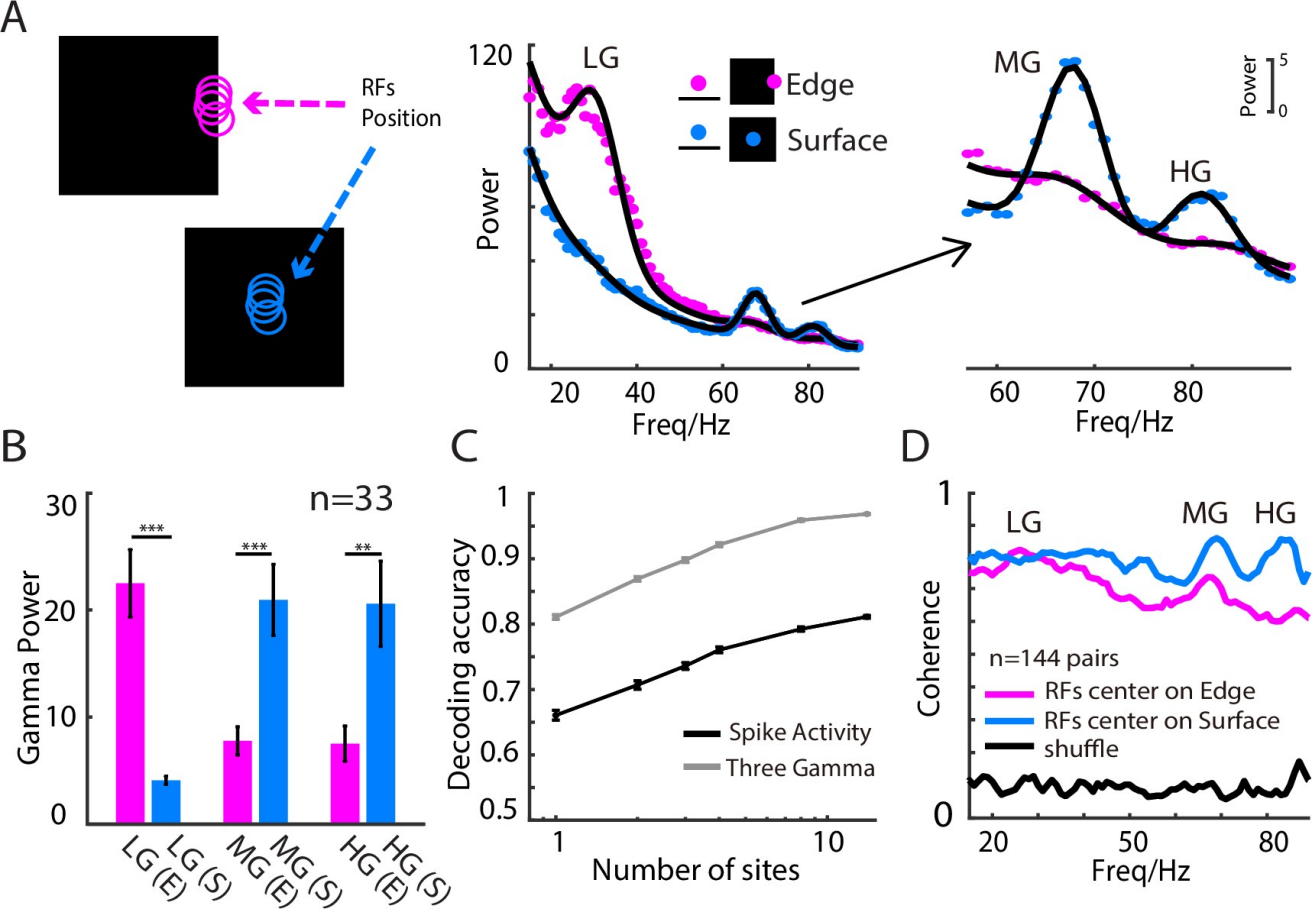
Three gamma rhythms have different preferences to surface/edge of uniform stimuli. The left panel in A shows an example of the visual stimuli: a large uniform black square shown in different positions (surface or edge) relative to the RF. The right panels in A show examples of power spectrums in the “RF on surface” and “RF on edge” conditions for a recording site (the blue curve denotes the edge condition, and the red curve denotes the surface condition). B shows a statistical comparison of the response strength in the “RF on surface” and “RF on edge” conditions for the 3 gamma components (33 sites in 2 animals). C shows that the decoding accuracy for the RF position (surface or edge) increased with the number of sites included (the grey curve represents the outcome when the model was trained with gamma power in the LG, MG, and HG bands, while the black curve shows that for the mean firing rate in 3 time bins). D shows the field–field coherence among recording sites with RFs on the square edge (purple) or square surface (blue). The panels A, C, and D are from one cat. The dataset underlying this figure can be found in https://github.com/CleveHan/Gamma-Oscillation. HG, high gamma; LG, low gamma; MG, medium gamma; RF, receptive field.

The results given in the previous sections indicate that 3 distinct GCs carry different SF information about grating patches. We further tested whether different SF information in a static object could also induce the 3 GCs. We presented a black or white square (25 degrees of visual angle, against a gray background) with uniform luminance for 2 seconds in different positions on the screen. The square was presented such that the RF of a site was either on the square’s edge, which had a higher SF information, or on its uniform surface (Fig 3A, left panel shows the black square condition), which contained low SF information. We found that LG emerged when the site’s RF was on the edge, while MG and HG emerged when the site’s RF was on the surface (Fig 3A, middle and right panels). Across the 3 gamma rhythms, the power of LG was significantly higher (*p* < 0.0001) at the edge (“RF on edge”) than at the surface (“RF on surface”) of the stimulus, while the power of MG (*p* < 0.0001) and HG (*p* < 0.01) was significantly higher for the stimulus surface (Fig 3B). Similar results were found in the white square condition (S6 Fig), but with weaker LFP gamma power (*p* < 0.0001) compared with the black square condition [22,23,54].

The results in the previous section indicate that the SF resolution of the 3 GCs in a recorded site might be better than that of the mean spike activity. We further tested this idea by decoding the RF positions (on the surface or edge of a simple square) of a site based on the gamma power or spiking activities of that site (see Materials and methods for details). We found that the 3 GCs together could not only be used to predict whether the RF of a site would be on the edge or surface of a simple square, (decoding accuracy was 81.1% ± 0.4%) but also had better decoding accuracy than that using the MUA (decoding accuracy was 66.3% ± 0.8%) from the same recorded site (see Fig 3C for the result from one animal and S7 Fig for that from another animal). Interestingly, the decoding performance of the 3 GCs from one recording site was even better than the decoding performance based on spike activity from multiple recording sites (Fig 3C) in our dataset. This suggests that the 3 GCs recorded from a single site might be a synchronized signal that pools SF information from spike activity occurring in many sites.

We further calculated the LFP coherence between 2 sites with RFs that were either both on the edge or both on the surface of a square with uniform luminance. We found a coherence peak in the LG band when the RFs of the sites were both on the square edge. By contrast, we found a coherence peak in the HG band when the RFs of the sites were both on the square surface (Fig 3D, S8 Fig). Interestingly, coherence peaks in the MG band could be seen for both edge and surface conditions. In brief, we have shown that 3 distinct gamma rhythms not only carry and synchronize different SF-related features for simple objects, but that they also discriminate SF-related features better than spiking activity, given a limited number of recording sites.

### Cortical distribution and possible origins of the 3 gamma rhythms

We next asked whether the 3 distinct gamma rhythms had same neural generation mechanisms. The correlation between the SF tuning properties (cutoff SF and SF selectivity) of gamma activation and that of the MUA (Fig 2E and 2F) suggest that the 3 GCs may have different neural originations. In this section, we directly measured gamma rhythms at different cortical depths and in the LGN.

**Fig 4:**
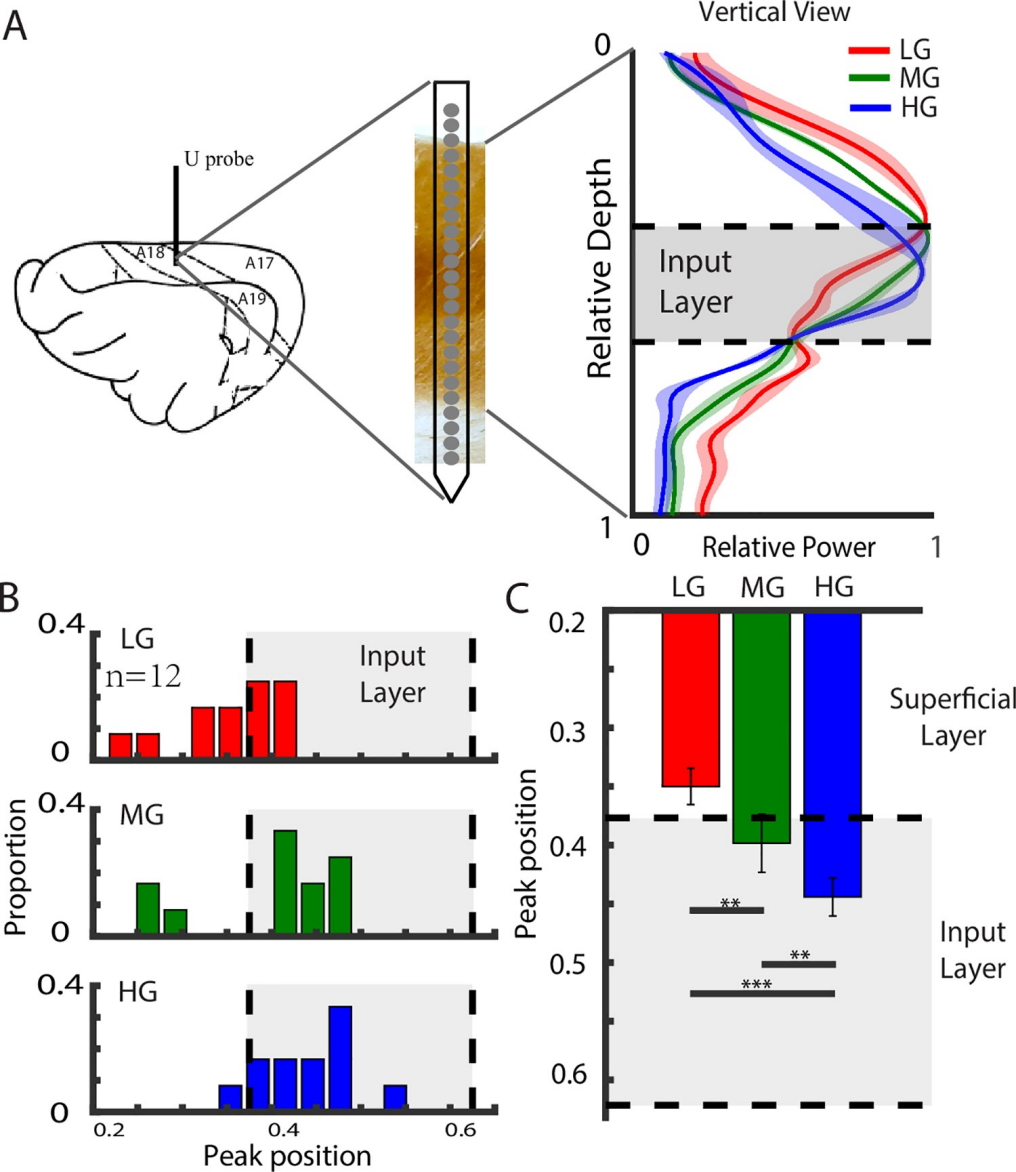
Different spatial distributions of gamma rhythms in V1. The left side of A shows an example of a laminar recording in V1, while the right side shows the power distributions in the vertical direction of V1 for the 3 gamma components in a typical linear array (shading indicates SEM). B is the distribution histogram of the peak positions of the 3 gamma components. C shows the comparison of the peak locations for the LG, MG, and HG (12 recording sessions in 3 cats, ** *p* < 0.01, *** *p* < 0.001, bootstrapping test). The dataset underlying this figure can be found in https://github.com/CleveHan/Gamma-Oscillation. HG, high gamma; LG, low gamma; MG, medium gamma; V1, primary visual cortex.

We used linear arrays (U-Probe) to simultaneously record from multiple sites across different layers (vertical direction) in V1 (Fig 4A). We found that the power of the 3 gamma rhythms peaked at different cortical depths (see the right side of Fig 4A for an example). There was a tendency for the HG power to peak in the input layer, the MG power to peak near the upper bound of the input layer, and the LG power to peak above the upper bound of the input layer (right side of Fig 4A). The cortical depths of the 3 gamma peaks were significantly different for the mean values from 3 animals (Fig 4B). The average peak of the LG was located in the superficial layer, that of the HG was in the input layer, and that for the MG fell between the other 2 components (*p* < 0.005 for all pair-wise comparisons) (Fig 4C).

**Fig 5:**
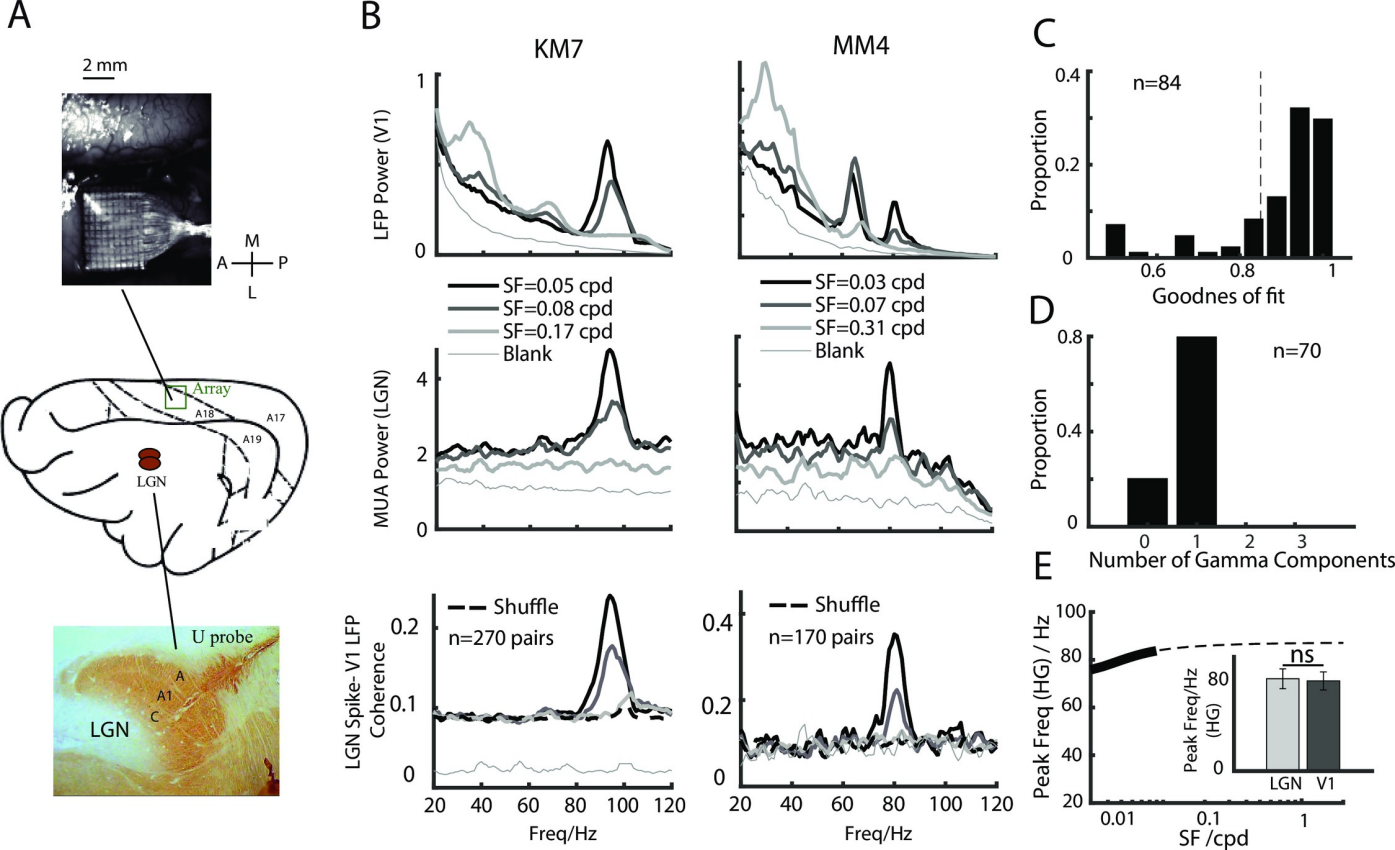
Different origins of gamma rhythms. A shows brain region recorded simultaneously by using a Utah array (in V1) and a linear array (in LGN). The top 2 rows of B show the LFP power spectrum in V1 and the MUA power spectrum in the LGN for different SFs, simultaneously recorded, in 2 cats. The bottom row shows the coherence between the MUA in the LGN and the LFP in V1 (the shuffled result is shown as a dashed line). C shows the distribution of the goodness of fit of this model for the MUA power spectrum of all of the recording sites in the LGN (8 placements in 6 cats, simultaneous V1 and LGN recordings in 4 cats, LGN only in 2 cats). D shows the proportion of the number of gamma components at each recording site. E shows the SF tuning curve for the peak frequency of the HG component. The thick line denotes frequency range estimated from out data. The small panel contains a bar graph that compares the peak frequencies of HG components simultaneously recorded in the LGN (*n* = 32) and V1 (*n* = 273). The dataset underlying this figure can be found in https://github.com/CleveHan/Gamma-Oscillation. HG, high gamma; LGN, lateral geniculate nucleus; MUA, multiunit activity; SF, spatial frequency; V1, primary visual cortex.

The positions of the power peaks of the HG and MG components in the input layer suggest that they may have subcortical origins. We next asked whether the HG and MG components originate in the visual thalamus. We used different arrays to simultaneously record from V1 (Utah array) and the LGN (U-Probe) (Fig 5A). Fig 5B shows the results of 2 typical simultaneous recordings: 3 gamma rhythms were found in V1 LFP signals (Fig 5B, top row), whereas only HG was found in the LGN MUA signals (Fig 5B, middle row). The SFC between the LGN and V1 (see Materials and methods) further indicated that only HG was generated in the LGN (Fig 5B, bottom row). Among the 84 recorded sites in the LGN, 83.3% of the MUA spectrum could be well fitted by the model (goodness of fit > 0.8, Fig 5C), and all of the well-fitted examples had only 1 GC in the high frequency range (Fig 5D, S9 Fig). The frequency ranges of the HG components were comparable between the LGN and V1 signals in the input layer (Fig 5E), indicating that the HG signal in V1 is likely inherited from the LGN. We observed a trend in which SF selectivity for HG in V1 was larger than that in the LGN (S10 Fig), as well as a trend in which the cutoff SF for HG in V1 was lower than that in the LGN. These results suggest that cortical inhibition might occur to modify the SF tuning transform from the LGN to V1 [45,46].

## Discussion

**Fig 6:**
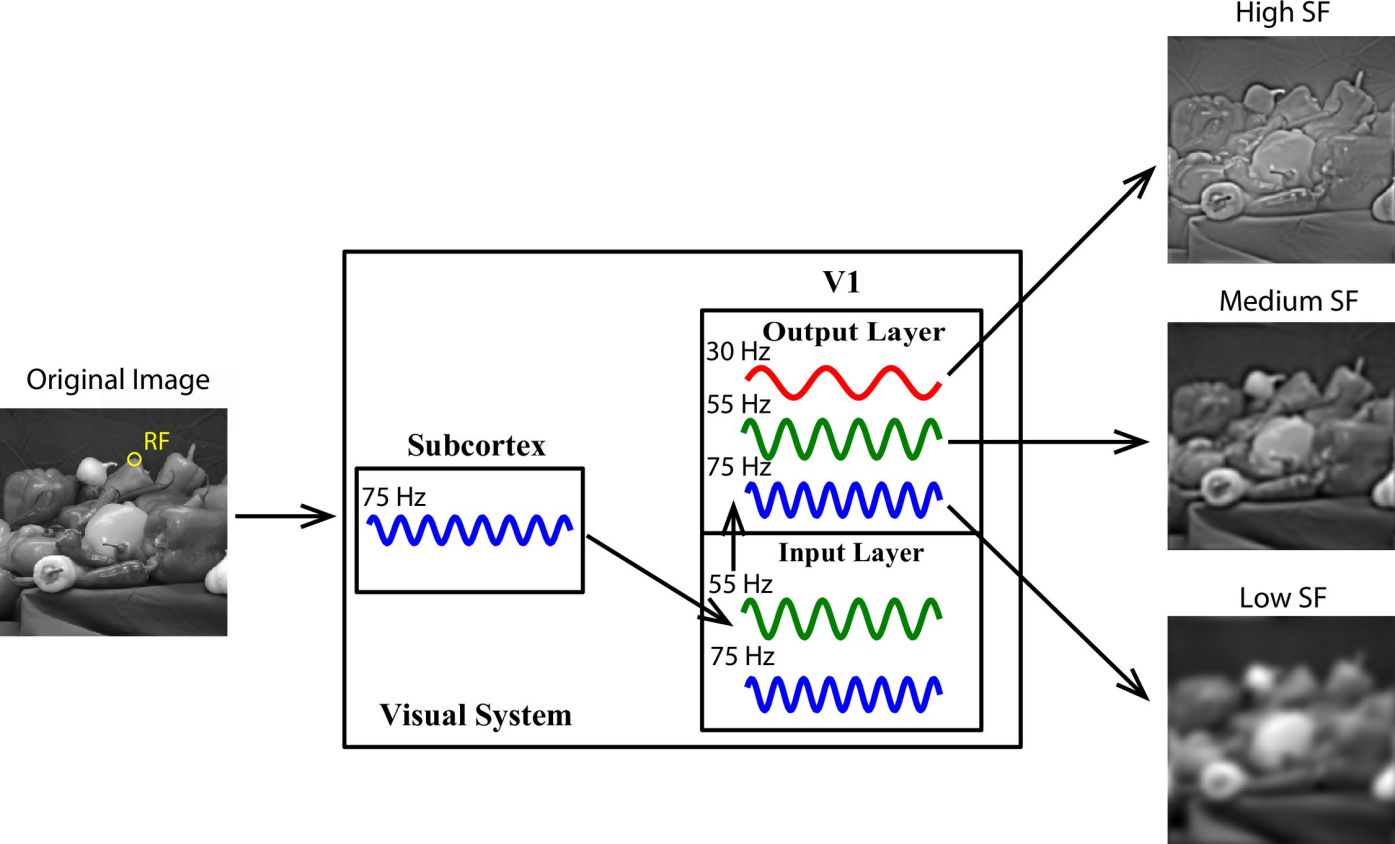
A schematic illustration of our main findings regarding the response properties and generation mechanisms of the 3 gamma rhythms. When the SF information from the natural images (see left) enters the visual system through the retina, to the SF information might be dissected into 3 different frequency channels (right column) in the superficial layer of V1. Signals in 3 colors (red, green, and blue) denote the 3 gamma components (LG, MG, and HG), respectively. HG, high gamma; LG, low gamma; MG, medium gamma; SF, spatial frequency; V1, primary visual cortex.

To our knowledge, this study is the first to report the presence of 3 narrowband gamma rhythms (distinct from the broadband gamma in previous studies [32,36,37,55–57]) with distinct response properties in the V1. The 3 gamma rhythms were distinguished by their oscillatory frequencies: “low gamma” (LG, approximately 25 to 40 Hz), “medium gamma” (MG, approximately 45 to 60 Hz), and “high gamma” (HG, approximately 65 to 90 Hz). The different GCs carried different aspects of visual information in terms of SF. Their distinct neural origins further suggest that gamma rhythms can be independently generated by multiple dynamical systems. Our findings are summarized in Fig 6.

### Neural mechanisms for distinct gamma rhythms

An important finding of this study is that the 3 detected gamma rhythms were generated by different neural circuits. Visually induced gamma band activity is generally thought to reflect network properties of excitatory and inhibitory interactions (E-I), which may exist at many stages of visual system (such as in the retina, LGN, and V1). To date, 2 mechanisms have been proposed regarding the generation of gamma rhythms in the visual cortex. Some studies have suggested that gamma is an emergent property of the cortex [58] and that it is likely to be generated in the superficial layer of V1 [59–62]. Other studies have proposed that gamma is generated in subcortical regions including the LGN [55,63] and retina [56]. Our results show that the HG component is generated in the subcortical region and that the generation of LG and MG is caused by cortical mechanisms (Fig 5). These findings reconcile contradicting mechanisms in related previous studies [20,32,33,35,55,56,64].

Our results further show that the MG component was strongest in the V1 input layer although the LG peaked in the V1 output layer (Fig 4). This suggests that MG might be related to local E-I in the input layer [65–73], while LG might be related to horizontal connections in the output layer, which has long-distance connections on a larger spatial scale. The distinct mechanisms of LG and MG are consistent with the model prediction from a recent computational study [34]. The involvement of different neural circuits in V1 gamma rhythms also explains the finding that each gamma rhythm has a specific relationship to the spike activity (S5 Fig) and suggests that different gamma bands might be related to different neural functions.

Besides the neural mechanisms of gamma rhythms at the circuit level, many studies have suggested that gamma may play a feedforward role in communication among different brain regions [62,63,67,74,75]. The bottom-up (feedforward input) or top-down (endogenous state) mechanism may also affect the property of gamma rhythms. Many studies have shown that gamma rhythms can be modulated by stimulus characteristics such as luminance/color [23,24,55,56,76,77], contrast [49,66,78], orientation [35,79–83], TF [33,36], stimulus size [32,36,66,84,85], and the complexity of visual stimuli such as noise masking grating [57,86,87], natural images [37,80,88], and superimposed gratings [64,89,90]. Our study provides systematic information regarding the SF of gamma rhythms. Some studies have also shown that gamma can be modulated by endogenous state, such as attention [10] or memory [8]. Since our experiment involved anesthetized animals, our results regarding the properties of gamma are more closely related to stimulus properties than to endogenous mechanisms.

### What is the functional role of gamma rhythms?

Our results indicate that both high [14–16,18–21] and low [22–24] SFs can induce strong gamma band activity. Specifically, these data represent the first evidence that different GCs encode specific SF information and that they might synchronize different features. More importantly, we found that the SF selectivity of LG was better than that of the mean firing rate. Accordingly, we propose a potential function for GCs in the visual cortex: Multiplexing SF-specific information synchronized [91–97] in distinct gamma bands. This proposal might not be contradictory to existing theories.

The 2 current hypotheses about gammas are the “binding by synchronization” (BBS) hypothesis [18,98–105] and the “communication by phase locking” (communication through coherence, CTC) hypothesis [2,106]. The “BBS” hypothesis emphasizes the integration of visual information about an object via gamma synchronization. However, the fact that SF information about a visual stimulus is carried by different GCs suggests gamma rhythms do not integrate visual features together in the visual cortex, but instead integrate them separately in different frequency bands. Through our coherence analysis, we found strong mutual synchronization between LG activities at recording sites with edge inside their RFs and strong mutual synchronization between HG activities at sites with RFs inside the square stimuli (S8 Fig). These results suggest that although SF-specific information is represented in different frequency bands, the gamma rhythms in each frequency band may still contribute to a routing-by-synchrony scheme.

The “CTC” hypothesis emphasizes the idea that information might be carried by different phases of a single gamma rhythm and that phase locking might be used for information integration in downstream cortical regions. Our findings suggest that visual information might be carried by different rhythms. Strong coherence between LGN and V1 in the HG band in our study supports the CTC theory. Our study also showed that there are spike–LFP phase locking for the low and medium GCs in V1. According to previous studies on 2 gammas in V1 [33,34], the spatial scale of the communication for LG and MG might be different. LG generated in superficial layer of V1 might have a relative larger communication scale than that of MG. But the exact forms of communication in these multiple gamma bands in downstream brain regions should be the focus of future theoretical and experimental studies.

There are 2 possible mechanisms for SF dependence in distinct gamma rhythms. The first is that there is generally a negative correlation between brain signals at different TF bands and their preferences for SFs, and the second is that the SF dependences of the 3 gamma rhythms reflect the characteristics of different neural circuits [34,59,66] that generate them. We verified that SF tuning of gamma rhythms is a property of the oscillatory components instead of the TF (S11 and S12 Figs).

### How many gamma rhythms are in the visual cortex?

We found 3 distinct narrowband GCs (LG, MG, and HG), although previous studies have only reported 1 or 2 gamma rhythms in the visual cortex of multiple species [9,18,20,23,33,62,79,81,107–109]. This discrepancy is likely due to the fact that gamma rhythms are dependent on the stimulus SF, and that most studies [18,20,79] did not vary the visual stimuli in a range of SFs. Because the 3 GCs had distinct SF tunings (Figs 1 and 2), stimulus conditions that could induce 3 simultaneous and significant GCs were relatively rare (the third row in Fig 1B). Our results also show that 1 or 2 obvious gamma rhythms could be seen under most stimulus conditions, although this could be seen as 3 gamma rhythms if the response patterns of the GCs are considered as a function of SF (Fig 1C). Another possibility why the 3 GCs might not have been reported previously could be the small size of the stimuli used in past studies [82,85]. Larger visual stimuli could induce stronger gamma rhythms compared with smaller visual stimuli [33,34,66]. The use of large visual stimuli in the present study allowed us to locate multiple significant gamma peaks.

Finally, in some high SF conditions, the LG may peak at around 25 Hz. According to the classical division of different frequency bands (beta: 14 to 25 Hz and gamma: 25 to 100 Hz), the rhythm in 25 Hz may be considered a beta rhythm instead of a GC. However, the peak frequency of the LG is a function of SF that is continuously monotonically decreasing (red curve in Fig 2B) from 35 to 25 Hz; hence, LG oscillates in the frequency of the gamma range. The identification of a type of rhythm should be considered using more experimental conditions and not simply judged by its TF.

## Supporting information

S1 Fig SNR of 3 detected gamma rhythms. A–C show the distributions of the SNR for 3 GCs in the recording sites that had a good model fit (goodness of fit > 0.8). D shows the distributions of the SNR for MUA. GC, gamma component; MUA, multiunit activity; SNR, signal-to-noise ratio.
(EPS)

S2 Fig Presence of 3 gamma rhythms in the power spectrum of MUA. The power spectrum of MUA for 2 example sites in 2 cats for different SFs, obtained by averaging the spectrum of the near SFs in each SF condition to decrease the noise. SF, spatial frequency; MUA, multiunit activity.
(EPS)

S3 Fig Explaining the power spectrum of MUA using a two-dimensional descriptive model. A shows the distribution of the goodness of fit for this model for all recording sites. B–E show the distributions of the SNR for the MUA and the 3 GCs in the recording sites that had good fit (goodness of fit > 0.8). F shows the proportion of the number of GCs in each recording site. GC, gamma component; MUA, multiunit activity; SNR, signal-to-noise ratio.
(EPS)

S4 Fig Distinct SF selectivity of the gamma rhythm in LFPs (for sites with at least 1 good gamma SNR). A shows the SF tuning curves for the LFP power of 3 GCs (red curve for LG, green curve for MG, and blue curve for HG). The dashed lines in B show the SF tuning curves for the peak frequency of the 3 GCs. The thick lines represent the estimated frequency that covered by our data (red curve for LG, green curve for MG, and blue curve for HG). C and D show bar graphs of SF selectivity and cutoff SF for the 3 GCs and MUA, respectively. GC, gamma component; LFP, local field potential; MUA, multiunit activity; SF, spatial frequency; SNR, signal-to-noise ratio.
(EPS)

S5 Fig Phase locking strength between LFPs and local neural response. We calculated the phase locking strength between LFPs from 1 to 120 Hz and the local neural response (*n* = 437). We used the Hilbert transform to filter the data to calculate the phase in each frequency band for different SFs. The firing rate in each LFP phase is shown in the same scale in the top row. The content of the second row is the same as that in the first row but with separate scales. The third row shows the phase at peak firing rate for different frequencies with different SFs. The fourth row shows the phase locking index, defined as (max firing rate − mean firing rate)/mean firing rate in different frequencies. The bottom row shows the normalized phase locking index (subtraction of the phase locking index in different SFs from the phase locking index in the blank condition). LFP, local field potential; SF, spatial frequency.
(EPS)

S6 Fig Weak gamma rhythms induced by a white surface. A comparison of the response strength in the “response frequency on surface” and “response frequency on edge” conditions with white polarity for the 3 GCs. The range of the y-axis is consistent with that in Fig 3B. GC, gamma component.
(EPS)

S7 Fig Performance (test accuracy) of the decoder in 2 cats. A shows the structure of the location decoder. B shows that the decoding accuracy in 2 cats (MM4 and NM1) for the position (surface or edge) increased with the number of sites included (the result shown as a gray curve was trained with gamma power in the LG, MG, and HG bands, and the result shown as a black curve was trained with the mean firing rates divided into 3 time bins). The decoders were trained with both black and white responses. Error bars indicate the standard error of the mean. HG, high gamma; LG, low gamma; MG, medium gamma.
(EPS)

S8 Fig Coherence between sites with both RFs inside a square or on the edges of the square. The left and right sides show the field–field coherence among recording sites with response frequencies corresponding to the square stimuli edge (red) or surface (blue) in 2 animals, respectively. RF, receptive field.
(EPS)

S9 Fig Explaining the power spectra of MUA in the LGN using a two-dimensional descriptive model. A–C show the distribution of the SNR for 3 GCs in recording sites that had good fit (goodness of fit > 0.8). GC, gamma component; LGN, lateral geniculate nucleus; MUA, multiunit activity; SNR, signal-to-noise ratio.
(EPS)

S10 Fig Comparison of SF selectivity and cutoff between the V1 and LGN. The left side shows a comparison of SF selectivity between the V1 (black bar) and the LGN (gray bar). The right panel shows the cutoff SF. LGN, lateral geniculate nucleus; SF, spatial frequency; V1, primary visual cortex.
(EPS)

S11 Fig SF tuning curve for baseline power and selectivity. A shows the SF tuning curves for the baseline power in the frequency band of the 3 GCs (red curve for LG, green curve for MG, and blue curve for HG). The bar graph of SF selectivity and cutoff SF for the 3 baseline power values for the corresponding frequency bands of GCs are shown in B and C, respectively. GC, gamma component; SF, spatial frequency.
(EPS)

S12 Fig Baseline power induced by white and black surface and edges. A shows a statistical comparison of the response strength in the “response frequency on surface” and “response frequency on edge” conditions with black polarity for the 3 baseline power values of the corresponding frequency bands of GCs. B shows the surface–edge ratio for the 3 baseline power values with black polarity. C shows a statistical comparison of the response strength in the “response frequency on surface” and “response frequency on edge” conditions with white polarity for the 3 baseline power values of the corresponding frequency bands of GCs. D shows the surface–edge ratio for the 3 baseline power values with white polarity. The range of the y-axis in A and C is consistent with that in Fig 3B and that in B and D is consistent with that in Fig 3C. GC, gamma component.
(EPS)

## Abbreviations

BBS: binding by synchronization
CRT: cathode-ray tube
CTC: communication through coherence
GC: gamma component
HG: high gamma
LFP: local field potential
LG: low gamma
LGN: lateral geniculate nucleus
MG: medium gamma
MSE: mean square error
MUA: multiunit activity
ReD: relative depth
RF: receptive field
SF: spatial frequency
SFC: spike-field coherence
SNR: signal-to-noise ratio
TF: temporal frequency
V1: primary visual cortex
